# An Audit of Metabolic Monitoring Compliance in Patients Initiated on Antipsychotics Across General Adult Wards in the East Midlands

**DOI:** 10.1192/bjo.2025.10685

**Published:** 2025-06-20

**Authors:** Sue Fen Tan, Isabel Hurren, Ranjit S. Badhan, Farina Tahira

**Affiliations:** 1 Nottinghamshire Healthcare NHS Foundation Trust, Nottingham, United Kingdom; 2 University of Nottingham, Nottingham, United Kingdom; 3 University Hospitals of Derby and Burton, Derbyshire, United Kingdom; 4 Derbyshire Healthcare NHS Foundation Trust, Derby, United Kingdom

## Abstract

**Aims:** The Lester Tool mandates baseline monitoring parameters for patients starting new antipsychotics or having their current antipsychotic regimen changed. These parameters include blood pressure, haemoglobin A1c (HbA1c)/fasting plasma glucose, lipids, lifestyle review, waist circumference, and weight, along with weekly weight monitoring for six weeks consecutively. This audit was initiated in response to concerns about rapid weight gain observed in many patients after starting certain antipsychotics. It aims to assess compliance with the Lester Tool to address the potential risks of metabolic syndrome in these patients. The audit seeks to understand the pattern of antipsychotic prescriptions as a secondary objective.

**Methods:** The audit was registered and ethically approved by the local research and audit department. A retrospective review of electronic health records and medication charts was conducted for 38 patients residing in two male and two female inpatient wards in the East Midlands between 17 June 2024 and 26 June 2024. Baseline parameters were audited to determine if they were measured within one week of antipsychotic initiation, and weekly weight checks thereafter. Waist circumference measurement at baseline was excluded due to concerns about its potential impact on patient self-esteem.

**Results:** Among baseline monitoring parameters, blood pressure had the highest compliance at 89.5%, followed by HbA1c/glucose (65.8%), lipids (57.9%), lifestyle review (55.3%), and weight monitoring (36.8%). Weekly weight follow-up compliance was low, with only 5.9% of patients meeting 100% compliance, and 41.2% of patients having no documented weight follow-up within six weeks. Non-compliance reasons were poorly documented. Risperidone was the most prescribed antipsychotic (N=9), followed by olanzapine (N=8), zuclopenthixol (N=7), and quetiapine (N=6). Olanzapine and risperidone were most frequently initiated in male wards, while zuclopenthixol and quetiapine were more common in female wards.

**Conclusion:** The audit identified significant gaps in compliance with the Lester Tool, which poses a risk to patients’ physical health due to the metabolic side effects of antipsychotic medications. The findings underscore the need for better documentation and communication regarding baseline and follow-up measures. Recommendations include increasing awareness of baseline blood requirements during admission, improving electronic health record functions (e.g. alerts for weekly weight checks and a drop-down to document weight check refusals), and enhancing coordination in monitoring patient weight following planned home leaves. A re-audit is ideal once the recommendations have been implemented.

